# Opening the Black Box: The Impacts of Environmental Regulations on Technological Innovation

**DOI:** 10.3390/ijerph17124365

**Published:** 2020-06-18

**Authors:** Muyao Li, Jinsong Zhang, Ramakrishnan Ramanathan, Ruiqian Li

**Affiliations:** 1 Accounting School, Harbin University of Commerce, Harbin 150028, China; 300268@hrbcu.edu.cn; 2 Business and Management Research Institute, University of Bedfordshire, Luton LU2 8LE, UK; ram.ramanathan@beds.ac.uk; 3 School of Economics and Business Administration, Heilongjiang University, Harbin 150080, China; liruiqian@hlju.edu.cn

**Keywords:** environmental regulations, technological innovation efficiency, technology investment efficiency, technology transformation efficiency

## Abstract

Environmental regulations (ERs) that can stimulate technological innovation (TI) are the key to enabling a win-win strategy that benefits both economic development and environmental protection. This study seeks to analyze the impacts of ERs on TI. Previous literature highlighted that the black box of TI can be decomposed into technology investment and technology transformation, but empirical studies on such a decomposition have largely been ignored. Moreover, a detailed discussion of the links between ERs and the decomposed components of TI has not been conducted in developing countries such as China. Our study attempts to address these research gaps by (i) decomposing TI using a novel data envelopment analysis (DEA) procedure and further analyzing the impacts of ERs on the decomposed components of TI and (ii) applying this novel methodology to Chinese context. Accordingly, this study is conducted in two stages. First, a novel application of the slack-based measure Network DEA model is developed to uncover the black box of TI using Chinese data in order to estimate the overall efficiency of technological innovation (TIE) and to decompose it into the efficiency of technology investment (TVE) and the efficiency of technology transformation (TTE). Second, a random effect Tobit model is applied to (i) investigate both the linear and nonlinear impacts of ERs on TIE in all sectors and (ii) examine whether the impacts of ERs on TVE and TTE in different subprocesses are heterogeneous or not. Our results have showed the benefits of decomposing TI: while technology transformation in China closely follows the trend of TI, the trend of technology investment is somewhat different. The estimation results further indicate that the impacts of ERs on TIE are nonlinear. Besides, ERs have heterogeneous impacts on the decomposed components of TI. The impacts of ERs on TVE are nonlinear, whereas the impacts of ERs on TTE are statistically insignificant.

## 1. Introduction

Academics and governments have been paying increased attention to the balance between economic development and environmental protection [1,2]. China is not an exception to this trend. In the past several decades, China has made tremendous economic achievements. However, environmental deterioration has become another serious problem due to the accelerated development of the economy [3,4]. To address environmental deterioration, a series of environmental regulations (ERs) have been enacted by the Chinese government in recent years [5,6]. The central government of China had implemented 30 state laws and 1400 industrial environmental standards by 2013 [7]. By 2017, local governments had implemented 480 local laws and 314 local rules and regulations related to environmental governance.

However, from a theoretical perspective, it remains unclear whether ERs can address environmental issues effectively or not and whether ERs enable a win-win strategy between economic growth and environmental protection [8]. Traditional wisdom believes that environmental protection and economic development are contradictory [9]. In contrast to traditional wisdom, the Porter Hypothesis claims that properly designed ERs can encourage firms to innovate both their products and production process. Technological innovation (TI) can help offset the compliance costs, enhance the utilization efficiency of resources and achieve competitive advantages [10,11]. Under this circumstance, the win-win situation can be achieved. Hence, determining whether ERs can stimulate TI or not is the key for enabling the win-win situation between economic development and environmental protection [12,13].

The impacts of ERs on TI have been widely studied for several decades [14,15], and diverse resulting relationships (e.g., positive, negative, neutral and nonlinear) have been found [14,16,17,18]. In this paper, we also analyze the impacts of ERs on TI in the Chinese context, but there are several differences compared to prior studies.

First, a comprehensive literature survey shows that most of previous studies treat TI as a single production process [19,20]. In reality, the whole process of TI can be more complex. According to the innovation value chain, TI can be divided into two related subprocesses, namely technology investment and technology transformation [15,19]. To match the reality more closely, this study thus decomposes TI into technology investment and technology transformation. Decomposing TI can help open the black box and see the inefficient subprocess of TI.

Second, when investigating the impacts of ERs on TI, the majority of extant studies generally employ a single indicator, such as research and development (R&D) investment and the number of patents, to capture TI [19]. However, only using one indicator to capture TI may cause bias, and TI can be better captured with multiple indicators [21]. Hence, we use multiple indicators to measure TI in this study. Besides, we show a novel application of the weighted slack-based measure (SBM) Network data envelopment analysis (DEA) model (hereafter called Network SBM DEA model) to evaluate the efficiency of TI. The Network SBM DEA model is a nonradial method that is suitable for inputs and outputs that do not change proportionally. The Network SBM DEA model can also take the importance of a subprocess into consideration by setting different weights to different subprocesses exogenously [22]. Hence, the Network SBM DEA model has some advantages in measuring the efficiency, but it is seldom employed to measure the overall efficiency of TI along with the efficiencies of its subprocesses in the existing studies. By employing the Network SBM DEA model, we not only evaluate the overall efficiency of TI (TIE) but also evaluate its subprocesses’ efficiencies, including the efficiency of technology investment (TVE) and the efficiency of technology transformation (TTE). In sum, our innovative application of the Network SBM DEA model to measure the overall efficiency of TI along with the efficiencies of its subprocesses is a significant contribution. 

Third, as highlighted earlier, the influence of ERs on TI has been widely examined for several decades. However, the existing studies fail to examine whether or not the impacts of ERs on different subprocesses of TI are heterogeneous [19]. As we divide TI into technology investment and technology transformation, apart from investigating the linear and nonlinear impacts of ERs on TI, we also examine whether the impacts of ERs on the decomposed components of TI are heterogeneous or not. In this regard, this study can further open the black box and provide a new perspective for understanding the effects of ERs on TI. 

Overall, the purposes of this study are to (i) estimate the overall efficiency of TI along with the efficiencies of two subprocesses of TI (TVE and TTE), (ii) explore both the linear and nonlinear ERs–TI links and (iii) explore whether the impacts of ERs on different subprocesses of TI are heterogeneous or not. 

By doing so, several contributions have been made. First, we open the black box by decomposing the whole process of TI into two different subprocesses rather than treating it as a single process. This can help identify the inefficient subprocess of TI. Second, we use multiple indicators instead of a single indicator to capture TI, and we employ a novel application of a Network SBM DEA model to measure the overall efficiency of TI and the efficiencies of its subprocesses. Third, we further open the black box by exploring whether the impacts of ERs on different subprocesses of TI are heterogeneous or not, which can provide a new perspective for understanding the link between ERs and TI. Finally, the novel examination of the impacts of ERs on TIE, TVE and TTE in the Chinese context is another contribution of this paper. 

## 2. Literature Review

The literature review section is divided into four sections. The first section focuses on Chinese context on regulations and innovation. Section 2.2 focuses on the decomposition of TI, and Section 2.3 focuses on the link between ERs and TI. The last section focuses on the application of DEA and Network DEA models.

### 2.1. A Brief Overview of Regulations and Innovation in the Chinese Context

China is a vast country with a number of environmental regulations and innovation regimes. To resolve the environmental problems caused by economic development, the Chinese government has made great efforts in environmental governance of industrial pollution. The government has established a sound environmental governance system with multiple actors implementing multiple environmental policy instruments that target polluting industries (or firms) [7]. Besides, the government has tightened its ERs in recent years. Some examples include the promulgation of the Air Pollution Prevention and Control Action Plan in 2013, the newly implemented policy of Environmental Protection Admonishing Talk in 2014, the amendment of Environmental Law in 2015 and the newly revised Air Pollution Prevention and Control Law in 2015 [23]. Overall, the tightened ERs can force firms to invest more in innovation so as to further improve environmental quality.

TI plays a crucial role in the coordinated development between economic development and environmental protection [12,13]. Hence, it is important for firms to improve their innovation capability and efficiency. To encourage firms to pay more attention to TI, the Chinese government has enacted a series of policies such as the National Medium- and Long-Term Plan for the Development of Science and Technology (2006–2020) in 2006, the Innovation-driven Development Strategy in 2012 and the Made in China 2025 government-led initiative in 2016 [24]. Through TI, traditional industries can be upgraded with advanced applicable technologies. This can not only help reduce energy consumption and adverse impacts on the environment, but also change the traditional development model of excessive resource consumption and environmental pollution.

### 2.2. Decomposing Technological Innovation into Different Subprocesses

TI is usually treated as a single production process in the existing literature [19,20]. Under this circumstance, the whole process of TI is regarded as a black box. However, the real TI production process can be more complex. According to innovation value chain, firms usually transform the knowledge they obtain into different technologies and products from which they generate revenue [25]. In this sense, TI can be decomposed into two parts. The first part relates to the activities that firms conduct to seek inputs for innovation [26]. The second part relates to the transformation of knowledge acquired by the firms into innovation outputs, which is also called the process of knowledge transformation [27]. Accordingly, Chen et al. [25] decompose innovation activities into the R&D process and the commercialization process. The R&D process applies the obtained knowledge to innovation, whereas the commercialization process introduces innovation into the market. Li et al. [19] decompose TI into technology investment and technology transformation. Zhu et al. [15] argue that TI can be decomposed into two stages: knowledge innovation and product innovation. Extant literature suggests the need for decomposing TI into different subprocesses from the perspective of the innovation value chain. 

### 2.3. The Link between Environmental Regulations and Technological Innovation

#### 2.3.1. The Linear Link between Environmental Regulations and Technological Innovation

##### Positive, Negative and Neutral Links

The direct linear link between ERs and TI has been a constant focus among scholars, especially in the early literature. Most conclusions support the Porter Hypothesis and suggest that ERs can stimulate TI.

A positive link between ERs and TI has been found in different countries, including India [28,29], Chile [30], Germany [31], Japan [32,33,34] and China [35]. Apart from TI, scholars also argue that ERs can also stimulate environmental innovation [36,37,38]. The positive influence of ERs on eco-innovation has been found in the US [39], Germany [40] and China [41,42,43,44]. Besides, Johnstone et al. [45] use unbalanced panel data obtained from 77 countries during 2001–2007 and conclude that ERs have positive impacts on environment-related innovation. Calel and Dechezleprêtre [46] find that the European Union Emissions Trading System contributes to low-carbon patenting and does not have a crowding-out effect on patents in regulated firms. 

In addition to the positive link, some scholars also prove that the link can be negative, as the compliance costs of ERs can crowd out the expenditure on TI [21,47]. Hence, the Porter Hypothesis is not supported. Brunnermeier and Cohen [48] confirm that the current laws and regulatory regimes do not provide incentives for firms to carry out TI in US manufacturing industries. Similarly, Chintrakarn [49] also argues that ERs have positive and significant impacts on technical inefficiency for the US manufacturing sector. For the neutral link, Smith and Crotty [50] confirm that the environmental policy called EU End of Life Vehicles Directive has limited influence on product innovation. Li et al. [19] find that ERs have no impact on the efficiency of TI. Yi et al. [51] show that the instruments of ERs employed in China do not favor green innovation. 

##### Uncertain Link

Some scholars also look beneath the surface and investigate some other factors that may affect the focal link. The results provide evidence for a more complex and uncertain relationship between ERs and TI, as the focal link can be affected by the organization’s structural flexibility and production process flexibility [52], the levels of firms’ innovation [53], and the extent of market uncertainty [54]. 

In addition, some scholars also distinguish ERs and innovation into different types and further demonstrate that the relationships between different kinds of ERs and TI [8,55,56] and the links between ERs and different types of innovation [57,58] are heterogeneous. Hence, both the types of ERs and the types of TI can affect the focal link.

#### 2.3.2. The Nonlinear Link between Environmental Regulations and Technological Innovation

Since there have been no consistent conclusions, scholars have further investigated the potential nonlinear link. 

The ever-increasing stringency of ERs can increase the compliance costs [59,60]. According to Restraint Theory, firms generally have limited resources and the ever-increasing compliance costs can crowd out firm’s R&D investment [3,61]. Therefore, ERs may inhibit TI in the short run. Nevertheless, the ever-increasing intensity of ERs may also provide incentives for firms to invest more in innovation in the long run. The increasing investments in innovation may generate extra profits so that the compliance costs may be partially or completely offset. Meanwhile, firms’ innovation capabilities can improve as well [62]. Thus, with the increasing of ER stringency, the effects of ERs on TI can shift from negative to positive from the perspective of dynamic development [63]. In summary, the link between ERs and TI can be U-shaped. Yuan et al. [64] show that the impact of ERs on TI is U-shaped in the subsample consisting of medium eco-efficiency sectors.

According to the Porter Hypothesis, well-designed ERs can stimulate TI, which can help in achieving a win-win strategy between economic development and environmental protection [10,11]. However, drawing on the theory of the ‘too-much-of-a-good-thing’ (TMGT) effect, there exists a maximum value for the link between two constructs, after which the increase in the beneficial antecedents can result in the decrease in the outcomes. The reason for this is that the additional costs can exceed the generated benefits when the beneficial antecedents surpass a certain level. Hence, the link between two constructs is inverted U-shaped [65,66]. As such, the link between ERs and TI is also likely to be inverted U-shaped. There exists an optimum intensity of ERs, after which the increase in ER can lead to the decrease in TI. Perino and Requate [67] prove the nonlinear impact of policy stringency on broad technology adoption. Liu and Gong [62] also demonstrate that the link between ERs and green innovation capacity is inverted U-shaped. 

In sum, the link between ERs and TI can be either U-shaped or inverted U-shaped on the basis of different theories, which proves the possibility of the nonlinear link.

### 2.4. Applications of DEA and Network DEA to Capture Technological Innovation When Investigating the Impacts of Environmental Regulations on Technological Innovation

DEA is a well-known methodology for estimating the relative efficiency of decision making units (DMUs) via a series of inputs and outputs [68,69]. Generally, the objective of DEA would be to identify the DMU that has maximized outputs consuming minimal inputs. 

#### 2.4.1. Applications of DEA when Investigating the Impacts of Environmental Regulations on Technological Innovation

In the existing literature, TI is usually measured with a single indicator, like R&D investment or the number of patents. However, TI can be better captured with multiple indicators [21,70]. Hence, the DEA model is a suitable choice. 

Scholars also developed some new methodologies after the traditional DEA was proposed. When scholars examine the effects of ERs on TI, they also use the newly developed methodologies to evaluate the efficiency of TI. Feng et al. [16] measure the efficiency of green innovation via super-slack-based measure and demonstrate that the influence of ERs on the efficiency of green innovation is significantly negative. Zhu et al. [15] construct a game cross-efficiency model to measure technological innovation efficiency and indicate that the effect of mandatory regulation on technological innovation efficiency turns out to be insignificant, whereas voluntary regulation has positive effects at the provincial level. Deng et al. [20] evaluate regional innovation performance via super-efficiency DEA model and show that stringent ERs can help to enhance regional innovation performance. Although these studies have used the evaluation results via different DEA methodologies as proxies for TI, TI is still treated as a single process.

#### 2.4.2. Applications of Network DEA When Investigating the Impacts of Environmental Regulations on Technological Innovation 

As mentioned above, TI should be decomposed into different subprocesses. Hence, DEA is not suitable, as it treats a DMU as a single production process that transforms inputs into outputs [71]. Instead, Network DEA is more suitable, as it considers a DMU as a network of interrelated processes. Decomposing the production process into different interrelated processes can help open the black box and provide deeper insights into the sources of the inefficiency [71]. However, only a few scholars have adopted Network DEA to evaluate the efficiency of different subprocesses [19,25], and no scholar has further explored the heterogeneous impacts of ERs on different subprocesses of TI. 

**Figure 1:**
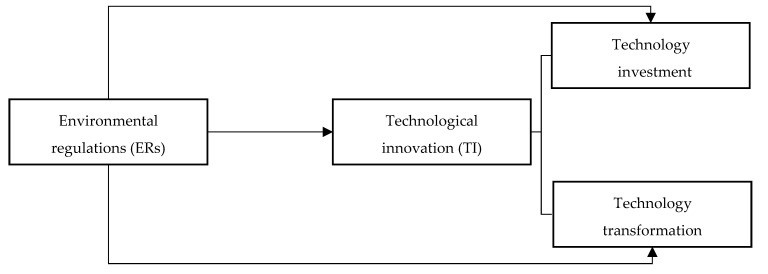
The conceptual framework of this study.

Overall, numerous studies have been conducted and no consensus has yet been reached. A systematic review of the literature indicates that decomposing TI into different subprocesses is more suitable, even though most scholars treat TI as a single process [15,16,20]. According to the literature on the link between ERs and TI, most scholars adopt a single indicator to capture TI [21]. Although some scholars also use the evaluation results via relevant DEA methodologies as proxies for TI, TI is still treated as a single process in most studies. Furthermore, the existing literature has neglected the heterogeneous impacts of ERs on the decomposed components of TI. Therefore, this study intends to enrich the investigation on the influence of ERs on TI in these aspects. Figure 1 presents the conceptual framework of our study.

## 3. Research Methods, Data and Analysis

We employed multiple research methods to conduct our analyses. For decomposing TI, we used a Network SBM DEA model. This is described in Section 3.1.1. We used a Tobit regression model to explore the link between ERs and TI. This model is described in more detail in Section 3.2.4.

### 3.1. Stage 1: Opening the Black Box—Decomposing the Efficiency of Technological Innovation Using Network DEA

**Figure 2:**
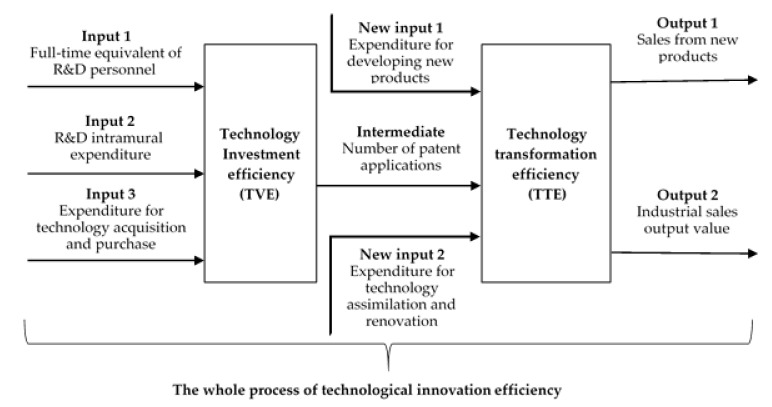
Decomposition of Technological Innovation.

Following Li et al. [19] and Chen et al. [25], we also divided TI into two related subprocesses according to the innovation value chain: technology investment and technology transformation. The objective of technology investment is to promote the progress of basic science and technologies, whereas the objective of technology transformation is to achieve and improve the commercial application of certain technologies produced in the former subprocess [25]. Figure 2 shows the decomposition of TI. The next section discusses the model, while the details of inputs, intermediates and final outputs for TI are included in Section 3.1.2.

#### 3.1.1. Network SBM DEA Model 

To operationalize the decomposition outlined in Figure 2, we adopted the weighted slack-based measure Network data envelopment analysis model (Network SBM DEA model) to measure the efficiencies of TI and its decomposed components [22,72]. The Network SBM DEA model has some merits in measuring efficiency, but it is seldom adopted to measure the efficiency of TI. By employing the Network SBM DEA model, the overall efficiency of TI (TIE) along with the efficiencies of two subprocesses (TVE and TTE) were measured in this study. More specifically, we utilized the input-oriented SBM under the variable returns-to-scale assumption with the fixed link case for evaluating the efficiencies of TI and its decomposed components. Besides, the weights of different subprocesses were needed during the calculation process of the overall efficiency. Both technology investment and technology transformation are equally important. Hence, the weights for technology investment and technology transformation were both set as 0.5. 

#### 3.1.2. Sample, Indicators and Data Sources

Our first objective was to estimate TIE, TVE and TTE of Chinese two-digit industrial sectors. The first two digits of the code represents the industrial sector to which the business belongs. From the total of 41 two-digit Chinese industrial sectors we included the 36 sectors described in Table 1 and collected data from between 2005 and 2015. Some sectors were not included because of missing data. More detailed information can be found in Table 1.

Following Chen et al. [25], Li et al. [19] and Zhu et al. [15], several indicators were adopted to evaluate TIE, TVE and TTE. For the measurement of TVE, the inputs included full-time equivalent of R&D personnel, R&D intramural expenditure and expenditure for technology acquisition and purchase. The output was the number of patent applications, which was also the input of technology transformation. For the measurement of TTE, the new inputs included expenditure for developing new products and expenditure for technology assimilation and renovation, whereas the final outputs were sales from new products and industrial sales output value. The data needed for this study were collected from *Statistics on Science and Technology Activities of Industrial Enterprises* and *China Industry Statistical Yearbook*. Table 2 shows the details of the indicators and the data sources of the indicators.

#### 3.1.3. Evaluation Results and Analysis

**Figure 3:**
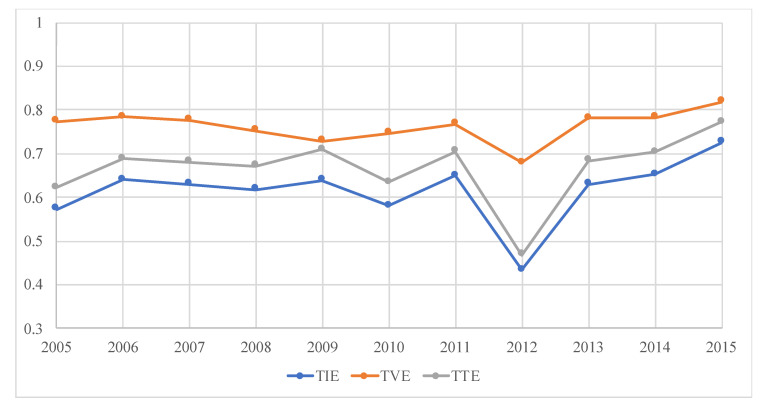
The variation of average efficiency scores over time. TIE, technological innovation efficiency; TVE, technology investment efficiency; TTE, technology transformation efficiency.

We employed MaxDEA 8 Ultra to evaluate the efficiency. While we do not show the annual efficiency score of each sector in this article, they are available upon request. The evaluation results of all sectors’ annual average TIE, TVE and TTE scores are displayed in Figure 3. From the holistic perspective, TIE, TVE and TTE increased slightly, although the efficiency score of each type fluctuated dramatically during the research period. The value of decomposition is clearly visible in Figure 3; while TTE seems to follow the same trend as TIE, the trend of TVE is somewhat different. First, TVE consistently recorded a high value, meaning that Chinese firms are very efficient in generating patents. Second, TVE showed a decreasing trend during 2006–2019 and an increasing trend in the subsequent two years, although TIE and TTE showed mixed trends during the same periods. Thus, opening the black box has revealed new insights into Chinese TI. Chinese industrial sectors currently place a heavy emphasis on technology investment rather than technology transformation. There is much room for the improvement of TTE.

### 3.2. Stage 2: Exploring the Impacts of ERs on TI

To further explore the impacts of ERs, we continued to use the sector-level data over the period of 2005 to 2015. 

#### 3.2.1. Dependent Variable: Technological Innovation

We carried out three different analyses to understand the impacts of ERs on (i) TI (without decomposition), for which the dependent variable is TIE; (ii) TVE; and (iii) TTE. All dependent variables were measured by the Network SBM DEA model described in Section 3.1.1.

#### 3.2.2. Independent Variable: Environmental Regulations

Scholars have adopted many different methods to measure ERs. The costs and expenditures of pollution abatement or pollutant discharge fees are frequently used methods [6,57,58,73]. Pollutant emissions are also used by scholars to represent ERs [74]. There are some other proxies employed by scholars, such as the number of newly implemented laws, regulations and rules [5,6]; the number of complaint letters on pollution and environment-related problems [6,7]; questionnaire survey results [75]; and the number of inspections [76]. 

Following Ye and Wang [8], Yuan et al. [64], Yuan and Xiang [21] and Liu and Xie [77], we used the operating costs of pollution treatment facilities, including industrial waste gas and industrial wastewater, to represent the stringency of ERs. The operating costs of industrial waste solid treatment were excluded due to the lack of data. Taking the influence of different scales into account, the operating costs of pollution treatment facilities were further divided by the industrial sales output value of each sector. The data required for ERs were collected from *China Industry Statistical Yearbook* and *China Statistical Yearbook on Environment* [78].

#### 3.2.3. Control Variables

We also incorporated a couple of control variables to ensure the quality of the empirical results according to extant literature. Table 3 describes the detailed information regarding the control variables. The required data for control variables were collected from *China Industry Statistical Yearbook.*

#### 3.2.4. Model Selection

Given that the range of the efficiency score was from 0 to 1, the random-effect Tobit model was used to conduct the analyses. The Tobit model can deal with a censored dependent variable [84]. 

To test the linear impacts of ERs, we constructed Model 1. Considering that it may take some time for ERs to exert their impacts on TI, ERs were lagged by one year [21,74]. Moreover, we also lagged all control variables by one year to eliminate the threat of endogeneity [5].

(1) $$TIE_{it}/TVE_{it}/TTE_{it}=C+\alpha_{1}ER_{it-1}+\alpha_{2}Size_{it-1}+\alpha_{3}Comp_{it-1}+\alpha_{4}D2A_{it-1}+\;\alpha_{5}FDI_{it-1}+\alpha_{6}Export_{it-1}+\epsilon_{it}$$

To test the nonlinear effects of ERs, we constructed Model 2. Compared with Model 1, we incorporated the quadratic term of ERs into the model. To reduce the potential threat of multi-collinearity, we centered the quadratic term before incorporating it into the models [85]. We also winsorized the continuous variables at the 1% and 99% levels to account for the bias caused by the outliers.

(2) $$TIE_{it}/TVE_{it}/TTE_{it}=C+\beta_{1}ER_{it-1}+\beta_{2}ER_{it-1}^{2}+\beta_{3}Size_{it-1}+\beta_{4}Comp_{it-1}+\;\beta_{5}D2A_{it-1}+\beta_{6}FDI_{it-1}+\beta_{7}Export_{it-1}+\epsilon_{it}$$

The other estimations of this study were conducted in the STATA 14.0 software. Table 4 shows the descriptive statistics of all variables. Because of missing data, the number of observations for ERs and foreign direct investment (FDI) were 393 and 391, respectively. The mean and median values of ERs were 33.04 and 18.36, showing a significant difference. The results indicated that regulation pressures that most firms face do not arrive at the average level. In addition, the mean values of TVE were the highest when compared with TIE and TTE.

Table 5 presents the correlation coefficients. The correlation coefficient between ERs and TIE was negative but not significant (β = −0.010, *p* = n.s.), whereas the correlation coefficient between ERs and TTE was also insignificant but positive (β = −0.026, *p* = n.s.). Conversely, ERs were significantly correlated with TVE (β = −0.103, *p* < 0.05). Although TIE, TVE and TTE were all significantly correlated with each other, they were dependent variables in separate models. 

We also examined the values of variance inflation factors (VIF) to check for problems with multi-collinearity. For all the regressions below, the maximum value of VIF was 3.87. Generally, if the values of VIF are under 10, multi-collinearity can be avoided [86]. Hence, multi-collinearity was not a problem in our estimation.

## 4. Empirical Results and Discussion

### 4.1. Regression Results

Table 6 shows the results of the influence of ERs on TI. The linear impacts of ERs on TIE are statistically insignificant (α = −0.0007, *p* = n.s.). However, the nonlinear impacts of ERs on TIE are significant, as the coefficient of (ER_st−1_)^2^ is significantly positive (β = 0.00002, *p* < 0.1). Together, the results prove that the impacts of ERs on TIE are nonlinear rather than linear.

In terms of the influence of ERs on the decomposed components of TI, the empirical results are different. Similar to the results of the influence of ERs on TIE, the impacts of ERs on TVE are also nonlinear. However, for the linear impacts of ERs on TTE, the regression results show a neutral type (α = −0.0007, *p* = n.s.), and the nonlinear regression results also present a neutral type (β = −0.0024, *p* < 0.1.; β = 0.00001, *p* = n.s.). It can be concluded that the effects of ERs on TTE are not statistically significant. Taken together, the results support that ERs have heterogeneous impacts on different subprocesses of TI.

### 4.2. Robustness Tests

In our model specification, ERs are lagged by one year. However, lagging ERs only one year may not account for the endogeneity sufficiently. To further address the reversed causality, following Leiter et al. [87], we incorporate two-year lagged ERs into the models and conduct the analyses again. Table 7 shows the relevant results. Except for the impacts of ERs on TTE, which show a U-shaped type, the rest of the results are similar with the results we have obtained for one-year lagged ERs. Besides, to address the endogeneity problem systematically, as suggested by Newey [88], we employ instrumental variable tobit to estimate the models again. We use the lagged ERs as the instrument [89]. The results are also shown in Table 7; they also confirm our previous findings. 

### 4.3. Discussion

Our results prove that ERs do not have linear impacts on TIE, TVE and TTE and the impacts of ERs on TIE are further from linear effects. The findings are consistent with some prior studies. Yuan et al. [64] show that the link between ERs and TI is U-shaped in the subsample consisting of medium eco-efficiency sectors. The nonlinear impacts of ERs on TIE prove the existence of a turning point. Since the quadratic term of ERs is positive, the negative impacts can be diminished with the increasing of the stringency of ERs. After surpassing the threshold, ERs can start to play a positive role in TI. In present-day China, the overall intensity of ERs is still not great enough to play a positive role, as the coefficient of ER_st−1_ is significantly negative (β = −0.0030, *p* < 0.05). Hence, the stringency of ERs should be improved continuously so that ERs can be beneficial to TI.

In addition, we open the black box by providing interesting results showing that ERs have heterogeneous impacts on different subprocesses of TI. The results strongly prove the need for the decomposition of TI and provide a novel perspective for understanding the effects of ERs on TI. The impacts of ERs on TVE and TTE changing from nonlinear to neutral suggests that the current intensity of ERs has different effects on technology investment and technology transformation. Under the same intensity of ERs, ERs can exert significant impacts on technology investment but exert insignificant impacts on technology transformation. Hence, to exert ERs’ significant and positive effects on technology transformation rather than only on technology investment, the stringency of ERs should be increased. Only when the intensity of ERs is strong enough can ERs have significant impacts on both technology investment and technology transformation. We also present a new finding showing that technology investment can be more sensitive than technology transformation as a response to ERs. A change in the increasing of the intensity of ERs may be able to exert their positive impacts on technology investment but not on technology transformation. 

Why should the intensity of ERs should be increased so that ERs can exert their significant impacts on technology transformation compared with technology investment? The reasons may lie in that technology investment and technology transformation represent different environmental strategies that firms adopt. When firms mainly focus on technology investment, they may respond to ERs sensitively but reactively. An increase in the intensity of ERs may quickly result in greater expenditure on technology investment [90]. Conversely, when firms focus more on technology transformation, they may start to adopt more proactive environmental strategies as a response to ERs. The aim of technology transformation is to generate revenue through the commercial application of certain technologies produced in the subprocess of technology investment [21,27,91]. Hence, when firms engage more in technology transformation and aim to achieve the commercial success of TI, they need to consider the demands of consumers and thus respond to ERs more proactively.

As mentioned earlier, sectors attach more emphasis on technology investment rather than technology transformation in present-day China (see Figure 3). Hence, how should firms be encouraged to adopt proactive environmental strategies rather than reactive environmental strategies? The key also lies in the intensity of ERs. With the increasing of the stringency of ERs, the environmental strategies that firms adopt may shift from reactive to proactive [82,92]. Hence, the stringency of ERs should be increased so that ERs can exert a positive effect on technology transformation. 

Our findings can help to deepen the understanding of the Porter Hypothesis. When firms engage more in technology transformation and not only in technology investment, the Porter Hypothesis can be better supported. When firms give priority to technology transformation, they start to adopt proactive rather than reactive environmental strategies so that TI can fully exert its positive effect on performance. When ERs are able to promote technology transformation, the win-win situations that benefit both the environment and the economy can be achieved more easily.

#### Policy Implications

To address environmental problems, the Chinese government has promulgated and enacted a series of policies aimed to improve the stringency of ERs and encourage firms to invest more in TI. To fully achieve the win-win strategy between economic development and environmental protection, drawing on our findings, we can propose some valuable implications for policy-makers. On one hand, the government needs to improve the intensity of ERs until the intensity can surpass the turning point so that ERs can start to exert positive effects on TI. On the other hand, under a certain intensity of ERs, ERs may exert their positive impacts on technology investment but not on technology transformation. Hence, the stringency of ERs should be increased so that ERs can exert positive impacts on technology transformation. In this way, ERs can exert positive impacts on two different components of TI. All in all, when the government improves the intensity of ERs, ERs can not only exert their positive impacts on all sectors, but also exert their positive impacts on different subprocesses of TI. 

## 5. Conclusions

Determining whether ERs can stimulate TI or not is the key for enabling a win-win strategy benefiting both economic development and environmental protection. In this study, we innovatively employ a Network SBM DEA model to estimate not only TIE but also TVE and TTE via dividing TI into two different subprocesses (technology investment and technology transformation). We further examine the linear and nonlinear links between ERs and TIE and examine whether ERs have varying impacts on TVE and TTE in order to help open the black box and see the heterogeneous impacts of ERs on different subprocesses of TI.

The data needed for the research questions come from different Chinese statistical yearbooks, and 36 sectors from 2005 to 2015 are included in our analyses. The evaluation results show that there is an overall increase in TIE, TVE and TTE. The importance of decomposing TI has been revealed, as it has been shown that the patterns of changes in TVE and TTE are different in China during the research period. The results further suggest that the impacts of ERs on TIE are nonlinear rather than linear. Besides, ERs have varying impacts on TVE and TTE. The impacts of ERs on TVE are nonlinear, whereas the impacts of ERs on TTE are statistically insignificant. On the basis of our results, we conclude that the intensity of ERs should be increased so that ERs can exert positive effects on technology transformation. 

This study enriches the existing studies in three aspects. First, we divide TI into two different subprocesses, rather than treating it as a single process, in order to open the black box and identify the inefficient subprocess of TI. Second, the majority of previous studies use a single indicator to represent TI and then investigate the impacts of ERs on TI. Instead, we use multiple indicators and innovatively use the evaluation results via the Network SBM DEA model to capture TI. Third, our study enhances the prior studies by analyzing the heterogeneous impacts of ERs on different subprocesses of TI in order to further open the black box and provide a novel perspective for understanding the effect of ERs on TI. Finally, the novel examination of the impacts of ERs on TIE, TVE and TTE in Chinese context is another contribution of this paper.

Despite of the contributions mentioned above, this study has some limitations as well. We believe future research can overcome these limitations. First, for the measurement of ERs, we could not include the operating costs of industrial waste solid treatment due to the unavailability of data. Future studies can consider these costs when available. Second, some scholars have proved that different types of ERs can exert varying influence on TI [15,55]. However, the data for different types of ERs on the sector level are not available. Future studies can investigate whether or not different types of ERs can exert varying influence on TI on the sector level when available. Third, future studies can research the moderation of industrial heterogeneity on the impacts of ERs on TI. Fourth, future studies can take regional heterogeneity into consideration and investigate whether the impacts of ERs on TI vary across different regions or not. Lastly, our study is conducted under the research setting of China. Given that China is the biggest developing country, this may limit the generalizability of the research findings [75]. Hence, scholars can collect data from other developed countries and verify the conclusions obtained in our study in their future research. 

## Figures and Tables

**Table 1:** Industrial sectors included in this study.

No.	Sectors	No.	Sectors
1	Mining and Washing of Coal	19	Manufacture of Raw Chemical Materials and Chemical Products
2	Extraction of Petroleum and Natural Gas	20	Manufacture of Medicines
3	Mining and Processing of Ferrous Metal Ores	21	Manufacture of Chemical Fibers
4	Mining and Processing of Nonferrous Metal Ores	22	Manufacture of Rubber and Plastic
5	Mining and Processing of Nonmetal Ores	23	Manufacture of Nonmetallic Mineral Products
6	Processing of Food from Agricultural Products	24	Smelting and Pressing of Ferrous Metals
7	Manufacture of Foods	25	Smelting and Pressing of Nonferrous Metals
8	Manufacture of Alcohol, Beverage and Refined Tea	26	Manufacture of Metal Products
9	Manufacture of Tobacco	27	Manufacture of General-Purpose Machinery
10	Manufacture of Textile	28	Manufacture of Special-Purpose Machinery
11	Manufacture of Textile and Apparel	29	Manufacture of Transportation Equipment
12	Manufacture of Leather, Fur, Feather and Related Products and Footwear	30	Manufacture of Electrical Machinery and Equipment
13	Processing of Timber, Manufacture of Wood, Bamboo, Rattan, Palm and Straw Products	31	Manufacture of Computers, Communications and Other Electronic Equipment
14	Manufacture of Furniture	32	Manufacture of Measuring Instruments
15	Manufacture of Paper and Paper Products	33	Other Manufacturing
16	Printing, Reproduction of Recording Media	34	Production and Supply of Electric Power and Heat Power
17	Manufacture of Articles for Culture, Education and Sport Activity	35	Production and Supply of Gas
18	Processing of Petroleum, Coking and Processing of Nuclear Fuel	36	Production and Supply of Water

**Table 2:** Indicators for evaluating the efficiency of Technological Innovation and data sources.

Technological Innovation		Indicators	Unit	Sources of Data
Technology investment	Inputs	Full-time equivalent of R&D personnel	Man-year	Statistics on Science and Technology Activities of Industrial Enterprises
		R&D intramural expenditure	10 thousand yuan	
		Expenditure for technology acquisition and purchase	10 thousand yuan	
	Intermediates	Number of patent applications	item	
Technology transformation	New inputs	Expenditure for developing new products	10 thousand yuan	
		Expenditure for technology assimilation and renovation	10 thousand yuan	
	Final outputs	New product sales	10 thousand yuan	
		Industrial sales output value	100 million yuan	China Industry Statistical Yearbook

**Table 3:** The details of control variables.

Variable	Name	Measurement	References
Industrial size	Size	Natural logarithm of total assets	[79]
Industrial competition	Comp	Natural logarithm of the number of firms	[80,81]
Debt ratio	D2A	Ratio of debt to assets	[82]
Foreign direct investment	FDI	Ratio of foreign direct investment to industrial sales output value	[5]
Export	Export	Ratio of export value to industrial sales output value	[83]

**Table 4:** Descriptive statistics of all variables.

Variable	*n*	Mean	S.D.	Median	Min	Max
Technological innovation efficiency (TIE)	396	0.615	0.347	0.503	0	1
Technological investment efficiency (TVE)	396	0.763	0.237	0.769	0.191	1
Technological transformation efficiency (TTE)	396	0.667	0.317	0.633	0.010	1
Environmental regulations (ERs)	393	33.04	40.32	18.36	0.711	196.5
Industrial size (Size)	396	8.723	1.216	8.759	6.273	11.14
Industrial competition (Comp)	396	6.763	1.080	6.896	3.970	8.447
Debt ratio (D2A)	396	0.544	0.080	0.560	0.239	0.681
Foreign direct investment (FDI)	391	0.249	0.166	0.246	0.001	0.814
Export	396	0.160	0.179	0.090	0	0.729

**Table 5:** Correlation coefficients of all variables.

Variable	TIE	TVE	TTE	ERs	Size	Comp	D2A	FDI	Export
TIE	1								
TVE	0.836 ***	1							
TTE	0.984 ***	0.731 ***	1						
ERs	−0.010	−0.103 **	0.026	1					
Size	−0.114 **	−0.128 **	−0.112 **	0.119 **	1				
Comp	−0.276 ***	−0.219 ***	−0.277 ***	−0.035	0.587 ***	1			
D2A	−0.142 ***	−0.166 ***	−0.121 **	0.179 ***	0.354 ***	0.465 ***	1		
FDI	0.060	0.142 ***	0.030	−0.341 ***	−0.120 **	0.279 ***	0.203 ***	1	
Export	0.101 **	0.186 ***	0.072	−0.346 ***	−0.264 ***	0.244 ***	0.115 **	0.807 ***	1

** *p* < 0.05, *** *p* < 0.01. TIE, technological innovation efficiency; TVE, technology investment efficiency; TTE, technology transformation efficiency; ERs, environmental regulations; Size, industrial size; Comp, industrial competition; D2A, debt ratio; FDI, foreign direct investment.

**Table 6:** Regression results.

Variable	TIE		TVE		TTE	
ERs_t−1_	−0.0007	−0.0030 **	−0.0004	−0.0031 ***	−0.0007	−0.0024 *
	(−0.86)	(−2.08)	(−0.70)	(−2.91)	(−0.92)	(−1.81)
(ERs_t−1_)^2^		0.00002 *		0.00002 ***		0.00001
		(1.93)		(3.06)		(1.58)
Size_t−1_	0.1636 **	0.1571 **	0.0806	0.0726	0.1597 **	0.1552 **
	(2.27)	(2.18)	(1.54)	(1.41)	(2.39)	(2.32)
Comp_t−1_	−0.2682 ***	−0.2865 ***	−0.1435 **	−0.1629 **	−0.2650 ***	−0.2794 ***
	(−2.89)	(−3.07)	(−2.16)	(−2.48)	(−3.08)	(−3.21)
D2A_t−1_	−0.5593	−0.4056	−0.5669	−0.3809	−0.4855	−0.3660
	(−0.87)	(−0.63)	(−1.19)	(−0.81)	(−0.82)	(−0.61)
FDI_t−1_	−0.1754	−0.2112	−0.1146	−0.1584	−0.2114	−0.2384
	(−0.28)	(−0.34)	(−0.26)	(−0.36)	(−0.37)	(−0.42)
Export_t−1_	0.3679	0.3680	0.1588	0.1596	0.4309	0.4293
	(0.69)	(0.70)	(0.41)	(0.42)	(0.88)	(0.88)
_cons	1.5151 ***	1.6638 ***	1.4950 ***	1.6525 ***	1.5264 ***	1.6378 ***
	(2.88)	(3.14)	(3.92)	(4.37)	(3.15)	(3.34)
N	353	353	353	353	353	353
Chi^2^	9.6474	13.3171	6.6694	16.1014	10.9326	13.2726

Z-statistics in parentheses; * *p* < 0.1, ** *p* < 0.05, *** *p* < 0.01. TIE, technological innovation efficiency; TVE, technology investment efficiency; TTE, technology transformation efficiency; ERs, environmental regulations; Size, industrial size; Comp, industrial competition; D2A, debt ratio; FDI, foreign direct investment.

**Table 7:** Regression results of robustness tests.

Variable	ERs Lagged Two Years			Variable	Instrumental Variable Tobit Regression		
	TIE	TVE	TTE		TIE	TVE	TTE
ERs_t−2_	−0.0042 ***	−0.0035 ***	−0.0036 ***	ERs_t−1_	−0.0039	−0.0054 ***	−0.0024
	(−2.89)	(−3.36)	(−2.64)		(−1.43)	(−2.89)	(−0.99)
(ERs_t−2_)^2^	0.00002 **	0.00002 ***	0.00002 *	(ERs_t−1_)^2^	0.0001*	0.0001 ***	0.00003
	(2.10)	(2.94)	(1.77)		(1.66)	(2.72)	(1.09)
Size_t−1_	0.1492 *	0.0798	0.1467 **	Size_t−1_	0.1735 ***	0.1125 ***	0.1524 ***
	(1.91)	(1.45)	(2.02)		(3.71)	(3.51)	(3.59)
Comp_t−1_	−0.2723 ***	−0.1534 **	−0.2679 ***	Comp_t−1_	−0.3383 ***	−0.2135 ***	−0.3051 ***
	(−2.86)	(−2.31)	(−3.00)		(−6.33)	(−5.87)	(−6.27)
D2A_t−1_	−0.6245	−0.4578	−0.5789	D2A_t−1_	−0.2529	−0.3228	−0.1316
	(−0.91)	(−0.92)	(−0.90)		(−0.45)	(−0.84)	(−0.26)
FDI_t−1_	−0.2467	−0.0915	−0.2809	FDI_t−1_	−0.2657	−0.1173	−0.2747
	(−0.38)	(−0.20)	(−0.47)		(−0.81)	(−0.52)	(−0.92)
Export_t−1_	0.3815	0.1493	0.4371	Export_t−1_	1.1529 ***	0.7569 ***	1.0389 ***
	(0.69)	(0.37)	(0.85)		(3.31)	(3.16)	(3.28)
_cons	1.7956 ***	1.5646 ***	1.7920 ***	_cons	1.6297 ***	1.4919 ***	1.5618 ***
	(3.21)	(3.98)	(3.45)		(5.31)	(7.06)	(5.60)
N	318	318	318	N	317	317	317
Chi^2^	17.7257	18.0344	17.2371	Chi^2^	48.4098	53.5856	46.2037

Z-statistics in parentheses; * *p* < 0.1, ** *p* < 0.05, *** *p* < 0.01. TIE, technological innovation efficiency; TVE, technology investment efficiency; TTE, technology transformation efficiency; ERs, environmental regulations; Size, industrial size; Comp, industrial competition; D2A, debt ratio; FDI, foreign direct investment.
